# The need to shift to a contextualized and collective mental health paradigm: learning from crisis-hit Lebanon

**DOI:** 10.1017/gmh.2020.20

**Published:** 2020-09-22

**Authors:** Tania Bosqui

**Affiliations:** Department of Psychology, American University of Beirut, P. O. Box 11-0236 Riad-el-Solh, Beirut 1107 2020, Lebanon

**Keywords:** Collective mental health, Social equality, Lebanon

The impact of national and international crises and disasters on population mental health has been well established, with poorer mental health and a higher incidence of mental disorders documented during war and armed conflict (Charlson *et al*., 2019), political instability and anti-government protests (Kai Hou *et al*., 2015), natural disasters (Makwana, 2019), economic crises (Bartoll *et al*., 2013), mass displacement (Morina *et al*., 2018), and linked more broadly to inequality, poverty and social injustices (Lund *et al*., 2010). Following the COVID-19 pandemic, mental health has been highlighted as a global priority in response to the fallout of the crisis, with the toll of lockdown, loss of jobs, increase in domestic violence, and lower social support impacting the wellbeing and functioning of affected populations (Holmes *et al*., 2020; UNHCR, 2020a; WHO, 2020). Research has shown that such crises, particularly when chronic, have an impact beyond the experience of the individual, affecting the functioning of families, communities, and wider social structures and socio-political systems (Sousa, 2013; Somasundaram, 2014). Despite major advances in the development and implementation of interventions in responding to crises and emergencies, there remain significant gaps in the evidence-base for public mental health responses that are sufficiently contextualized to this collective suffering.

## The collective in existing models of mental health and care

Psychological literature has tended to focus on singular experiences and associated individualized interventions, neglecting wider social systems and interpersonal processes (Maercker and Horn, 2013). Widely used psychological models of post-traumatic stress disorder (PTSD), for instance, have major conceptual limitations when applied in the context of humanitarian disasters, chronic instability, inequality, and social injustice. Such models have been developed for the most part in European or North American populations at the individual level. They generally assume a certain level of current safety, a clear distinction between pre- and post-trauma, and that hypervigilance and a sense of current threat are maladaptive symptoms linked to the over appraisal of danger and poor integration of trauma memories (Foa *et al*., 1989; Ehlers and Clark, 2000). They have limitations, therefore, in their application to populations facing ongoing and very real threats of violence and loss. Indeed, the application of psychological models of trauma to war affected populations has been heavily criticized because it risks imposing an individual approach inappropriate to the context of collective trauma (Wessells, 2009), and undermining natural and normal reactions to complex instability and violence (Summerfield, 2000).

Within displaced Palestinian populations, local idioms of distress that reflect collective suffering and social distress provide documentation of broader conceptualizations of mental health and coping. Words like *somoud* (strength in adversity or steadfastness) express the resilience of the collective, and words like *sadma* (shock), *faji'a* (tragedy), and *musiba* (calamity) communicate a range of collective trauma experiences (Giacaman *et al*., 2011). To attempt to apply individualist and medicalized binary categories of mental disorders to this collective distress so tightly intertwined with oppression and injustice, is to oversimplify mental health and to neglect contextualized and collective experience. Such individualized simplifications can also lead to missing other mechanisms that are key for understanding and mitigating psychological distress in context. Research in conflict-affected populations has consistently shown strong mitigating factors of interpersonal relationships (Bosqui *et al*., 2017), social support (Besser and Neria, 2012), collective cohesion and action (Greenley *et al*., 1975), and social equality and justice (Burns and Esterhuizen, 2008), through a fluid process of community resilience in which ‘cultural, political, and social factors interact in the face of adverse conditions’ (Nuwayid *et al*., 2011, p. 507). Community resilience is the cooperative and collective pooling of resources that enables coping, at the collective and individual levels, in the context of major events or changes, unpredictability, and uncertainty (Berkes and Ross, 2013; Somasundaram and Sivayokan, 2013). The mechanisms underlying community resilience are not included in traditional models of stress that are predominantly grounded in cognitive or behavioral theories, and tested in populations in relative safety and security. More recent socio-interpersonal models of PTSD have attempted to integrate social and individual processes into our understanding of trauma reactions (Maercker and Horn, 2013). It is imperative, more than ever during the current pandemic, that this integration of broader community, collective, and systemic perspectives on mental health is used to inform mental health and psychosocial support for crisis-affected populations.

The international guidelines for mental health and psychosocial support in humanitarian emergencies recommends a tiered model of care, which includes a strong focus on interventions at the basic services and security, family and community support, and focused psychosocial support levels, that is responsive to context and culture, and builds on existing resources (IASC, 2007). Specialized mental health care is only a small part of this model, dedicated to treating clinical disorders and delivered by trained professionals. The evidence-base however, remains thin at the broader levels of intervention, with the strongest evidence for specialist and focused service provision. A review of evidence found that specialized and focused psychosocial support in emergency and humanitarian settings was supported by multiple randomized control trials, while only a handful supported family and community support, and no trials were published on interventions at the basic services and security level (Tol *et al*., 2013). This means that the levels of intervention that reach the most people and address collective experiences are the least well established. These levels may include advocacy, community-led initiatives, preventative interventions, activating social networks, and communal traditional support (Nuwayid *et al*., 2011; Somasundaram, 2014). Even with the growing evidence for family and community support, many interventions remain limited to the diluted application of cognitive and behavioral models of intervention developed in non-crisis affected countries by specialist clinical providers (Bosqui and Marshoud, 2018), while multi-layered care systems that integrate community, family, and individual components are poorly supported (Tol *et al*., 2011; Betancourt *et al*., 2013). This is problematic because the current evidence-base neglects broader interpersonal processes and collective experiences that could be instrumental to mitigate harm. There is a clear need for the development and empirical testing of social change and community based interventions, that include family and community components, and that are contextualized, feasible, and sustainable in crisis settings.

## Learning from crisis-hit Lebanon

Lebanon is a middle-income country reeling from a dual explosion in the port of Beirut that ripped through the city killing hundreds, injuring thousands, and displacing hundreds of thousands. The blasts, known locally as the port explosions or disaster, the bombing of Beirut, or the Beirut massacre, were blamed on explosive materials, including more than 2700 tons of ammonium nitrate, improperly stored for years despite warnings of its lethal capacity. The explosions come at a time when the country was already facing an economic disaster of a scale not seen since the civil war. The economic crisis, linked to decades of state corruption, triggered a popular uprising, or *thawra* (revolution), in October 2019. Hundreds of thousands of people took to the street, united across sectarian lines to demand system reform, an end to corruption, and improved social equality and human rights. Lebanon is the third highest indebted country in the world, and has one of the highest income inequality distributions; 2% of the population earns the same as over 60% of the entire population (UNDP, 2017). In addition, Lebanon has been affected by a refugee crisis, with a government estimated 1.5 million refugees in a total population of around 5.5 million (UNHCR, 2020b). Refugees in Lebanon suffer from poor educational and economic opportunities, socio-economic insecurity, and widespread prejudice and discrimination (WHO, 2017). Other migrant groups brought to the country through the notorious *kafala* (sponsorship) system to work as domestic workers, face other challenges through restrictions of movement and personal freedoms, as well as exploitation and mistreatment by employers (Fernandez, 2018).

The painful impact of the worsening economic crisis in Lebanon has begun to take its toll on many residents, with loss in incomes and the closure of businesses widespread. Lebanon's unemployment rate has soared, with well over a third of the Lebanese population living below the poverty line (World Bank, 2020). For some refugee populations, this figure is closer to 90% (UNHCR, 2020b). The first COVID-19 positive patient was diagnosed in Lebanon in late February 2020, and the Lebanese government swiftly introduced social distancing measures by shutting down schools, universities, parks, shops, restaurants, and cinemas. With increasing cases of COVID-19, a whole country lockdown was introduced in mid-March 2020, including the closures of the airport, port, and borders, as well as strict rules on wearing masks outdoors and a nighttime curfew, enforced by a heavy police and army presence. The inability of daily workers to earn a salary, mixed with price increases through the sharp devaluation of the Lebanese Lira, and a severely limited social welfare system, put many residents at risk of life-threatening hardship (Devi, 2020). The Beirut port explosions have spiraled the country into further pain, and re-ignited anger over the corruption and incompetence of the ruling elite. The devastation of Beirut and its people, the COVID-19 pandemic, and the worsening economic crisis is a disaster for Lebanon on a scale that we have yet to comprehend. The socio-political, public health, and economic crises have and will lead to further losses and deteriorating quality of life – Lebanon is facing an extraordinary strain on individual, family, community, and national coping resources.

Collective suffering, and resilience, has been well documented throughout past crises in Lebanon. During the July 2006 war, collective experiences of the war were found to be significantly associated with anxiety symptoms, regardless of individual exposure (Yamout and Chaaya, 2011), and with action-orientated or risk taking behaviors (Karam *et al*., 2007). In the aftermath of the port explosions, action-oriented behaviors have been observed, with hundreds of volunteers from all over the country providing help to clear debris, cover broken windows and doors, and offer water, food and shelter to affected people (An-Nahar, 2020). The collective processing of events, through the sharing of stories, videos, and CCTV footage, has also been intensely observed in the days and weeks following the explosion. Experiences during the 2006 war were found to be associated not just with distress, but with community resilience, when such experiences were comprised of community cohesiveness, a shared identity, and social support. Adequate public health interventions and social solidarity were found to help sustain this resilience over time (Nuwayid *et al*., 2011). The current provision of mental health care in Lebanon is predominantly through the private health care system for which many low-income and marginalized people have no access (WHO, 2017). However, the Ministry of Public Health launched the National Mental Health Program in 2014, and a 5-year Mental Health and Substance Use Strategy with the aim of reforming the Mental Health System in line with WHO global action plan for Mental Health 2013–2020 (Karam *et al*., 2016). Much has been achieved, including training primary care workers, rolling out evidence-based treatments (De Almeida and Saraceno, 2018), and improving the integration and accessibility of services for Syrian refugees (El Chammay *et al*., 2016). The program has also supported research, such as projects that aim to improve access to the WHO's Problem Management Plus program using technology (Shehadeh *et al*., 2020), scale up similar programs for children and adolescents (Brown *et al*., 2019), and develop family focused psychosocial support for at-risk young people drawing on existing family and community knowledge (Bosqui *et al*., 2020). In response to the COVID-19 crisis a coordinated plan was developed with WHO and UNICEF, in line with IASC guidelines (NMHP, 2020a), that aimed to use the crisis as an opportunity to strengthen system reform grounded in community participation and human rights (El Chammay and Roberts, 2020). Then, in response to the Beirut port explosions, a national action plan was developed within days of the explosions, focusing on doing no harm and coordinating multiple local and international agencies responses to the overwhelming mental health needs of the population (NMHP, 2020b). These are strong examples of a public health approach that engages and supports communities, responds to changes in context, and attempts to address community or population level distress. Gaps remain however, in the evidence-base for family and community interventions, as well as at the level of basic services and security in the context of the current collective loss and community distress. The impact of the devastating explosions, economic crisis, and persistent government corruption and mismanagement, has created a palpable sense of chronic uncertainty, anger, and collective anxiety in the country. While major strides have been taken in improving access to evidence-based care, attention to the needs of the collective are imperative to mitigate the mental health consequences of the worst crises in Lebanon since the civil war.

## The need to shift to a contextualized and collective mental health paradigm

The snowballing crises and collective distress in Lebanon, serve to demonstrate the limitations of individualistic clinical models and the strengths of a community and systemic public health approach. Other countries affected by significant collective emergencies, crises or pervasive social inequalities, including high-income countries, could benefit from this altered approach. This is not to disregard neurobiological, genetic, cognitive, or behavioral influences on mental health, but to acknowledge the major impacts of social and economic context, and to incorporate this into our models of understanding, and public mental health responses. The current global COVID-19 pandemic is no exception, mental health services will grapple to function if they maintain provision at the individual level and neglect the wider ecological system levels of family, community, and country. We know that social adversity, injustice, inequality, poverty, and chronic stress exposure, are strongly linked to increased mental health difficulties (Lund *et al*., 2010), and correspondingly that social cohesion and community resilience can buffer the impact of national crises (Nuwayid *et al*., 2011). Yet, models of understanding, service structures, and interventions in many country contexts remain largely grounded in medical frameworks and individualized treatment. A shift to a contextualized and collective mental health paradigm can help to integrate evidence at every ecological level, and to respond to changes in context. The neglect of such an integrated approach, in theory and practice, can otherwise feed into, and replicate, tragic mental health and health inequalities. The shift to a contextualized and collective mental health paradigm, alongside improving the evidence-base and implementation of multi-layered systemic interventions, can help to address this tragedy, and inform a shift in current mental health service policy and provision during, and beyond, these crises.

## References

[ref1] An-Nahar (2020) Lebanese unite to assist victims following Beirut massive explosion. Available at https://en.annahar.com/article/1249853-lebanese-unite-to-assist-victims-following-beirut-massive-explosion (Accessed 18 August 2020).

[ref2] Bartoll X, Palencia L, Malmusi D, Suhrcke M and Borrell C (2013) The evolution of mental health in Spain during the economic crisis. European Journal of Public Health 24, 415–418. 24367067 10.1093/eurpub/ckt208

[ref3] Berkes F and Ross H (2013) Community resilience: toward an integrated approach. Society & Natural Resources: An International Journal 26, 5–20.

[ref4] Besser A and Neria Y (2012) When home isn't a safe haven: insecure attachment orientations, perceived social support, and PTSD symptoms among Israeli evacuees under missile threat. Psychological Trauma: Theory, Research, Practice, and Policy 4, 34–46.

[ref5] Betancourt TS, Meyers-Ohki S, Charrow AP and Tol WA (2013) Interventions for children affected by war: an ecological perspective on psychological support and mental health. Harvard Review of Psychiatry 21, 70–91. 23656831 10.1097/HRP.0b013e318283bf8f PMC4098699

[ref6] Bosqui T, Brown F, Jordans MJ, Betancourt T, Carr A, Donnelly M and Ghoissany, M (2020) The effectiveness, mechanisms of change, and acceptability of Family Focused PsychoSocial Support (FFPSS) for at-risk adolescents in Lebanon. Available at https://gtr.ukri.org/projects?ref=AH%2FT007419%2F1#/tabOverview (Accessed 4 July 2020).

[ref7] Bosqui T and Marshoud B (2018) Mechanisms of change for interventions aimed at improving the wellbeing, mental health and resilience of children and adolescents affected by war and armed conflict: a systematic review of reviews. Conflict and Health 12, 15. 29760768 10.1186/s13031-018-0153-1 PMC5941634

[ref8] Bosqui T, Marshoud B and Shannon C (2017) Attachment insecurity, post-traumatic stress and hostility in adolescents exposed to armed conflict. Peace and Conflict 23, 372–382.

[ref9] Brown FL, Steen F, Taha K, Aoun M, Bryant RA, Jordans MJD, Malik A, van Ommeren M, Abualhaija A, Ibrahim Said Aqel I S, Ghatasheh M, Habashneh R, Sijbrandij M, El Chammay R, Watts S, Akhtar A (2019) Early adolescent skills for emotions (EASE) intervention for the treatment of psychological distress in adolescents: study protocol for randomised controlled trials in Lebanon and Jordan. Trials 20, 545. 31477178 10.1186/s13063-019-3654-3 PMC6721330

[ref10] Burns JK and Esterhuizen T (2008) Poverty, inequality and the treated incidence of first-episode psychosis. Social Psychiatry and Psychiatric Epidemiology 43, 331–335. 18253683 10.1007/s00127-008-0308-2

[ref11] Charlson F, van Ommeren M, Flaxman A, Cornett J, Whiteford H and Saxena S (2019) New WHO prevalence estimates of mental disorders in conflict settings: a systematic review and meta-analysis. Lancet (London, England) 394, 240–248. 10.1016/S0140-6736(19)30934-1 PMC6657025 31200992

[ref12] De Almeida J and Saraceno B (2018) Mid-term Evaluation Report of the National Mental Health Strategy for the Lebanon MOPH National Mental Health Programme. Beirut: Ministry of Public Health.

[ref13] Devi S (2020) Economic crisis hits Lebanese health care. Lancet (London, England) 395, 548. 10.1016/S0140-6736(20)30407-4 32087781

[ref14] Ehlers A and Clark DM (2000) A cognitive model of posttraumatic stress disorder. Behavior Research and Therapy 38, 319–345. 10.1016/s0005-7967(99)00123-0 10761279

[ref15] El Chammay R, Karam E and Ammar W (2016) Mental health reform in Lebanon and the Syrian crisis. The Lancet 3, 203. 26946388 10.1016/S2215-0366(16)00055-9

[ref16] El Chammay R, Roberts B (2020) Using COVID-19 responses to help strengthen the mental health system in Lebanon. Psychological Trauma: Theory, Research, Practice, and Policy 12(S1), S281–S283. doi: 10.1037/tra0000732.avoid. 32538651

[ref17] Fernandez B (2018) Health inequities faced by Ethiopian migrant domestic workers in Lebanon. Health & Place 50, 154–161. 29454243 10.1016/j.healthplace.2018.01.008

[ref18] Foa EB, Steketee G and Rothbaum BO (1989) Behavioral/cognitive conceptualisation of post-traumatic stress disorder. Behavior Therapy 20, 155–176.

[ref19] Giacaman R, Rabaia Y, Nguyen-Gillham V, Batniji R, Punamäki R-L and Summerfield D (2011) Mental health, social distress and political oppression: the case of the occupied Palestinian territory. Global Public Health 6, 547–559. 21108104 10.1080/17441692.2010.528443

[ref20] Greenley JR, Gillespie DP and Lindenthal JJ (1975) A race riot's effect on psychological symptoms. Archives of General Psychiatry 32, 1189–1195. 1180669 10.1001/archpsyc.1975.01760270121017

[ref21] Holmes EA, O'Connor RC, Perry VH, Tracey I, Wesserly S, Arseneault L, Ballard C, Christensen H, Cohen Silver R, Everall I, Ford T, John A, Kabir T, King K, Madan I, Michie S, Przybylski AK, Shafran IR, Sweeney A, Worthman CM, Yardley L, Cowan K, Cope C, Hotopf M, Ed Bullmore E (2020) Multidisciplinary research priorities for the COVID-19 pandemic: a call for action for mental health science. The Lancet. Psychiatry 7(6), 547–560. doi: 1016/S2215-0366(20)30168-1. 32304649 10.1016/S2215-0366(20)30168-1 PMC7159850

[ref22] IASC (2007) IASC guidelines on Mental Health and Psychosocial Support in Emergency Settings. Geneva: World Health Organization Inter-agency Standing Committee.

[ref23] Kai Hou H, Hall BJ, Canetti D, Lau KM, Ng SM and Hobfoll SE (2015) Threat to democracy: physical and mental health impact of democracy movement in Hong Kong. Journal of Affective Disorders 186, 74–82. 26232750 10.1016/j.jad.2015.07.005 PMC7127225

[ref24] Karam E, El Chammay R, Richa S, Naja W, Fayyad J and Ammar W (2016) Lebanon: mental health system reform and the Syrian crisis. BJPsych International 13, 87–89. 29093915 10.1192/s2056474000001409 PMC5619489

[ref25] Karam E, Fayyad JA, Salamoun MM, Debs MM, Tabet CC, Karam AN, Cassir Y, , Cherfane J, Dimashkieh M, Dimassi H, Farah L, Hage R, Hajjar R, Mekhael L, Mneimneh Z, Sawaya L, Siriani N, Tanios C, Tcheurekjian M (2007) Assessment Study of Psychosocial Status of Children and Adolescents in the South of Lebanon and Southern Suburbs of Beirut after the July 06 War. Beirut: Institute for Development, Research, Advocacy and Applied Care.

[ref26] Lund C, Breen Am Flisher AJ, Kakuma R, Corrigall J, Joska JA, Swartz L and Patel V (2010) Poverty and common mental disorders in low and middle income countries: a systematic review. Social Science & Medicine 71, 517–528. 20621748 10.1016/j.socscimed.2010.04.027 PMC4991761

[ref27] Maercker A and Horn AB (2013) A socio-interpersonal perspective on PTSD: the case for environments and interpersonal processes. Clinical Psychology and Psychotherapy 20, 465–481. 22730216 10.1002/cpp.1805

[ref28] Makwana N (2019) Disaster and its impact on mental health: a narrative review. Journal of Family Medicine and Primary Care 8, 3090–3095. 10.4103/jfmpc.jfmpc_893_19 PMC6857396 31742125

[ref29] Morina N, Akhtar A, Barth J and Schnyder U (2018) Psychiatric disorders in refugees and internally displaced persons after forced displacement: a systematic review. Frontiers in Psychiatry 9, 1–15. 30298022 10.3389/fpsyt.2018.00433 PMC6160546

[ref30] NMHP (2020a) Action Plan MHPSS Response to the COVID-19 Outbreak in Lebanon-V1.0. Beirut: Ministry of Public Health.

[ref31] NMHP (2020b) National Action Plan for the MHPSS Response to Beirut Explosion Disaster. Beirut: Ministry of Public Health.

[ref32] Nuwayid I, Zurayk H, Yamout R and Cortas CS (2011) Summer 2006 war on Lebanon: a lesson in community resilience. Global Public Health 6, 505–519. 21424963 10.1080/17441692.2011.557666

[ref33] Shehadeh HMJ, Abi Ramia J, Cuijpers P, El Chammay R, Heim E, Kheir W, Saeed K, van Ommeren M, van’t Hof E, Watts S, Wenger A, Zoghbi E, Carswell K (2020) Step-by-step, an e-mental health intervention for depression: a mixed methods pilot study from Lebanon. Frontier Psychiatry 10, 986. 10.3389/fpsyt.2019.00986 PMC7034323 32116815

[ref34] Somasundaram D (2014) Addressing collective trauma: conceptualizations and interventions. Intervention 12, 43–60.

[ref35] Somasundaram D and Sivayokan S (2013) Rebuilding community resilience in a post-war context: developing insight and recommendations – a qualitative study in Northern Sri Lanka. International Journal of Mental Health Systems 7, 2–24. 23305538 10.1186/1752-4458-7-3 PMC3630062

[ref36] Sousa CA (2013) Political violence, collective functioning and health: a review of the literature. Medicine, Conflict and Survival 29, 169–197. 10.1080/13623699.2013.813109 PMC3801099 24133929

[ref37] Summerfield D (2000) Childhood, war, refugeedom and ‘trauma’: three core questions for mental health professionals. Transcultural Psychiatry 37, 417–433.

[ref38] Tol WA, Barbui C, Galappatti A, Silove D, Betancourt TS, Souza RS, Golaz A and van Ommeren M (2011) Mental health and psychosocial support in humanitarian settings: linking practice and research. Lancet (London, England) 378, 1581–1591. 10.1016/S0140-6736(11)61094-5 PMC3985411 22008428

[ref39] Tol WA, Bastin P, Jordans MJD, Minas H, Souza R, Weissbecker I, Van Ommeren M (2013) Mental health and psychosocial support in humanitarian settings, (eds. Patel V, Minas H, Cohen A and Prince MJ (eds), Global Mental Health: Principles and Practice, pp. 384–400. Oxford University Press: Oxford.

[ref40] UNDP (2017) Assessing Labor Income Inequality in Lebanon's Private Sector Findings, Comparative Analysis of Determinants, and Recommendations. Beirut: UNDP Fiscal Policy Advisory and Reform Project at the Lebanese Ministry of Finance.

[ref41] UNHCR (2020a) UNHCR urges prioritization of mental health support in coronavirus response. Available at https://www.unhcr.org/news/press/2020/5/5ebcfd784/unhcr-urges-prioritization-mental-health-support-coronavirus-response.html (Accessed 6 February 2020).

[ref42] UNHCR (2020b) Lebanon. Available at http://reporting.unhcr.org/node/2520 (Accessed 22 April 2020).

[ref43] Wessells MG (2009) Do no harm: toward contextually appropriate psychosocial support in international emergencies. American Psychologist 4(8), 842–854. 10.1037/0003-066X.64.8.842 19899908

[ref44] WHO (2017) WHO-AIMS Report on Mental Health System in Lebanon. Beirut: World Health Organization.

[ref45] WHO (2020) Mental Health and Psychosocial Considerations During the COVID-19 Outbreak. Geneva: World Health Organization.

[ref46] World Bank (2020) Lebanon Economic Monitor. Available at https://www.worldbank.org/en/country/lebanon/publication/lebanon-economic-monitor-fall-2019 (Accessed 11 April 2020).

[ref47] Yamout R and Chaaya M (2011) Individual and collective determinants of mental health during wartime. A survey of displaced populations amidst the July–August 2006 war in Lebanon. Global Public Health 6, 354–370. 20677034 10.1080/17441692.2010.494163

